# The use of laser in urogynaecology

**DOI:** 10.1007/s00192-018-3844-7

**Published:** 2018-12-18

**Authors:** Alka A. Bhide, Vik Khullar, Stephen Swift, Giuseppe A. Digesu

**Affiliations:** 1 St Mary’s Hospital, Imperial College NHS Trust, London, UK; 2 Medical University of South Carolina, Charleston, SC USA

**Keywords:** CO_2_ laser, Erbium laser, Urinary incontinence, Prolapse, Menopause

## Abstract

**Introduction:**

The use of lasers in urogynaecology has increased in recent years. Their use has been described in pelvic organ prolapse, urinary incontinence and genito-urinary symptoms of menopause. The aim of this study was to review the published literature on CO_2_ and erbium:YAG laser use in urogynaecological conditions.

**Methods:**

An extensive search of literature databases (PubMed, EMBASE) was performed for publications (full text and abstracts) written in English up to July 2018. Relevant trials were selected and analysed by an independent reviewer. Twenty-five studies were identified in total.

**Results:**

All studies were either prospective cohort or case-control studies. The results of individual studies indicate that both CO_2_ and erbium lasers are effective in treating urogynaecological conditions. Most studies use a vaginal approach with only two investigations of intraurethral application.

**Conclusion:**

The use of lasers to treat these conditions may seem appealing; however, the lack of good-quality evidence in the form of multi-centre randomised placebo-controlled trials is concerning. The safety and effectiveness of these laser devices have not been established. Use of lasers may lead to serious adverse events such as vaginal burns, scarring, dyspareunia and chronic pain. Randomised placebo-controlled trials in addition to formal evaluation of the laser devices are required before this treatment modality can be recommended.

## Introduction

Interventions in urogynaecology have been under the spotlight recently. Concerns about mesh and its adverse outcomes have led our speciality quite rightly to scrutinise the evidence behind the advice and treatments we offer to our patients. Robust evidence and clear guidance have never been more important when counselling women about their treatment choices. The use of lasers to treat urogynaecological conditions such as stress incontinence and pelvic organ prolapse seems to be increasing. The aim of this review is to assess the current published evidence of this treatment modality.

The estimated prevalence of urinary incontinence in middle-aged and older women in the general population is in the range of 30–60% [1]. It encompasses stress urinary incontinence (SUI), overactive bladder (OAB) and mixed urinary incontinence (MUI, both stress and OAB). Treatment options for stress incontinence include weight loss, pelvic floor exercises, medication and surgery [2].

Pelvic organ prolapse (POP) (the sensation of a mass bulging into the vagina) has a reported prevalence of between 5 and 10% in the general population [1]. Treatments include doing nothing, vaginal pessaries, pelvic floor exercises and surgery. Surgical options can involve pelvic floor repair using native tissue or biological grafts. The use of synthetic mesh for pelvic organ prolapse has been temporarily suspended in the UK by NHS England and undergone several restrictions in other countries over concerns about patient safety. Women also present with vaginal relaxation syndrome (VRS) where relaxing of the vaginal walls can lead to both physical and psychological distress, the main one being lessening of sexual satisfaction for both the female and her partner. This is often referred to as the vagina feeling ‘loose’ [3].

Genito-urinary syndrome of menopause (GSM) has been defined as including a range of symptoms related to a decline of circulating ovarian hormones, such as vaginal dryness, dyspareunia, recurrent urinary tract infections and urinary incontinence [4]. As these symptoms usually associated with menopause may affect sexual function and quality of life, women often seek treatment. Options include lubricants and moisturisers, which offer temporary relief, in addition to hormonal topical preparations for a more long-term effect. However, there are some women who do not wish to take hormonal preparations or for whom oestrogen is contraindicated.

The use of lasers in gynaecology has been reported in the literature [5]. The aim of this review is to look at the evidence available on the use of lasers to treat urinary incontinence, pelvic organ prolapse and genitourinary symptoms of menopause.

## Methods

An extensive search of literature databases (PubMed, EMBASE) was performed for publications (full texts and abstracts) written in English. Keywords included vaginal CO_2_ laser, vaginal YAG erbium laser, urinary incontinence, pelvic organ prolapse, vaginal relaxation syndrome, genito-urinary syndrome of menopause and vulvo-vaginal atrophy. Twenty-five studies were identified containing the above keywords and were included. These are summarised in Table 1.

**Table 1:** Summary of included studies

Author, date, [ref]	Number	Age (mean or median*)	Study design	Laser type	Condition	Evaluation	Assessment parameters
Gambacciani et al., 2015 [6]	19	60.9 ± 8.1	Prospective cohort	Vaginal erbium:YAG	SUI	Follow-up at 3 months post-treatment	ICIQ-UI SF
Ogrinc et al., 2015 [7]	175	49.7 ± 10	Prospective cohort	Vaginal erbium:YAG	MUI	Follow-up at 12 months	ICIQ-SF, ISI
Fistonic et al., 2016 [8]	31	46.6	Prospective cohort	Vaginal erbium:YAG	SUI	Follow-up at 1, 2 and 6 months	ICIQ-SF UI
Fistonic et al., 2016 [9]	73	47 (41–54)*	Prospective cohort	Vaginal erbium:YAG	SUI	Follow-up at 1, 2–6 months	ICIQ-SF UI
Pardo et al., 2016 [10]	42	46.5 (30–70)*	Prospective cohort	Vaginal erbium:YAG	SUI	Follow-up at 3–6 months	ICIQ-SF
Pitsouni et al., 2016 [11]	53	57.2 ± 5.4	Prospective cohort	CO_2_ laser	LUTS	Follow-up 3 months	ICIQ-FLUTS, ICIQ-UI SF, UDI, KHQ
Gaspar et al., 2017 [12]	22	57.9	Prospective cohort	Intra-urethral erbium:YAG	Type III stress incontinence	Follow-up at 3 and 6 months	ICIQ-UI SF
Gambacciani et al., 2018 [13]	114	64.6 ± 4.4	Prospective cohort	Vaginal erbium:YAG	SUI	Follow-up at 12, 18 and 24 months	ICIQ-SF
Perino et al., 2016 [14]	30	56*	Prospective cohort	CO_2_ laser	OAB	Follow-up at 1 month	OAB-Q SF
Lin et al., 2017 [15]	30	52.6 ± 8.8	Prospective cohort	Vaginal erbium:YAG	OAB	Follow-up at 3 and 12 months	OABSS
Gaspar et al., 2011 [5]	92	Not stated	Case control	CO_2_ laser, PRP and PFE vs. PRP and PFE	GSM	Pre- and post-treatment	Sexual health questionnaire, vaginal biopsies
Salvatore et al., 2014 [18]	49	59.6 ± 5.8	Prospective cohort	CO_2_ laser	GSM	Follow-up at 3 months	VHIS, VAS, SF-12
Salvatore et al., 2015 [19]	77	60.6 ± 6.2	Prospective cohort	CO_2_ laser	GSM	Follow-up at 12 months	FSFI, SF-12
Perino et al., 2015 [20]	48	56*	Prospective cohort	CO_2_ laser	GSM	Follow-up at 1 month	VHIS, VAS
Pitsouni et al., 2016 [11]**	53	57.2 ± 5.4	Prospective cohort	CO_2_ laser	GSM	Follow-up at 3 months	Vaginal maturation value VHIS, FSFI
Sokol et al., 2016 [21]	30	58.6 ± 8.8	Prospective cohort	CO_2_ laser	GSM	Follow-up at 3 and 12 months	VAS, VHIS, FSFI, SF-12
Behnia-Willison et al., 2017 [23]	102	61	Prospective cohort	CO_2_ laser	GSM	Follow-up at 2–4 and 12–24 months	Frequency and severity of GSM symptoms, Australian pelvic floor questionnaire
Gambacciani et al., 2015 [6]	62	Laser 60.9 ± 8.1 Estriol 63 ± 4.5	Case control	Vaginal erbium:YAG vs. 50 mcg estriol twice weekly for 3 months	GSM	Follow-up at 1, 3 and 6 months	VAS, VHIS
Gaspar et al., 2017 [24]	50	Laser 55 ± 6.7 Estriol 53.5 ± 5.7	Case control	Vaginal erbium:YAG vs. 0.5 mg estriol twice weekly for 3 months	GSM	Follow-up at 12 and 18 months	VAS of GSM symptoms
Gaspar et al., 2018 [25]	29	66	Prospective cohort	Intraurethral vaginal erbium:YAG	GSM (urinary symptoms)	Follow-up at 3 and 6 months	ICIQ-SF, 1-h pad test, VAS
Gambacciani et al., 2018 [13]	205	Laser 61.2 ± 7.2 Estril 62.0 ± 7.5	Case control	Vaginal erbium:YAG vs. 50 mg estriol gel twice weekly for 3 months/non-hormonal therapies	GSM	Follow-up at 12, 18 and 24 months	VAS, VHIS
Gaviria et al., 2012 [27]	21	37.7	Prospective cohort	Vaginal erbium:YAG	VRS	Follow-up at 3 months	Specific VRS questionnaire, POP-Q, PISQ-12
Lee 2014 [3]	30	41.7	Prospective cohort	Vaginal erbium:YAG	VRS	Follow-up at 2 months	Histology, perineometry, patient satisfaction
Bizjak-Ogrinc et al., 2013 [28]	28	56.1	Prospective cohort	Vaginal erbium:YAG	Prolapse	Follow-up at 2, 4 and 6 months	Clinical examination, post-void residual
Bizjak-Ogrinc et al., 2017 [29]	83	56.1	Prospective cohort	Vaginal erbium:YAG	Prolapse	Follow-up at 2, 6, 12, 24 and 36 months	Clinical discomfort VAS, patient satisfaction

**Study participants included those with LUTS and GSM

## Use of lasers in female stress incontinence

Gambacciani et al. [6] conducted a small study on 45 women with GSM, 19 of whom had SUI. Symptoms were evaluated at baseline and at 3-month follow-up with the ICIQ-UI SF questionnaire. Treatment involved the vaginal erbium:YAG laser (1 treatment every 30 days for 3 months). Laser treatment induced a significant decrease in the ICIQ-SF score of 12.0 ± 1.8 to 5.6 ± 2.6 at 3-month follow-up. These scores also remained significantly lower than baseline at the 6-month follow-up.

A larger study looked at 175 women with urinary incontinence to assess the vaginal erbium:YAG laser as a potential treatment strategy for SUI and MUI [7]. Women with newly diagnosed SUI and MUI (66% and 34%, respectively) were included. Incontinence was classified by type (SUI and MUI) and grade (mild, moderate, severe and very severe) using ICIQ-SF and assessing the Incontinence Severity Index (ISI). Altogether, one laser procedure was performed in 12 patients, 2 procedures in 54 patients and 3 procedures in 109 patients. At 1-year follow-up, ISI was significantly decreased in all women. One hundred eight patients from both SUI and MUI groups exhibited no incontinence at 1-year follow-up, 29 patients had unchanged urinary incontinence, and 38 patients noticed worsening of UI compared with baseline. In patients with SUI, UI significantly improved in 88% of the cases while in those with MUI only 34% improved. No major adverse effects were noticed in either group. This study demonstrated that at 1-year follow-up the vaginal erbium:YAG laser could be considered a potential treatment strategy for SUI but does not seem appropriate for treating MUI cases.

A small pilot study looked at the effect of a single vaginal erbium:YAG laser treatment with follow-up at 1, 2 and 6 months post-treatment. Thirty-one female patients with SUI were recruited and assessed using the ICIQ-UI. The degree of incontinence and its impact on quality of life were assessed using the ICIQ-UI SF. All patients were followed up at 1 month, 56.7% were followed up at 2 months and 43.4% remained in the study at the 6-month follow-up point. The ICIQ-UI score significantly decreased in all follow-ups compared with baseline. No serious adverse events were reported [8]. The same group conducted a larger prospective cohort single-centre study of 73 female patients suffering from stress urinary incontinence. The aim was to assess the outcome of laser treatment for mild-moderate SUI groups. Inclusion criteria were a history of vaginal delivery, stress urinary incontinence, normal cell cytology, negative urine culture, and a vaginal canal, introitus and vestibule free of injuries and bleeding. Each patient received one intravaginal laser erbium:YAG treatment with follow-up at 1 and 2–6 months post-intervention. The degree of incontinence and impact on quality of life were assessed with the ICIQ-UI SF questionnaire. From baseline to 2–6-month follow-up, 72.3% of patients experienced improvement, 23.4% experienced no change and 4.3% experienced worsening of symptoms based on the ICIQ-UI scores. No serious adverse events were noted. The authors concluded that use of the vaginal erbium:YAG laser resulted in a clinically relevant short-term improvement in stress urinary incontinence [9].

Pardo et al. evaluated the vaginal erbium:YAG laser in a longitudinal prospective study in 42 women with mild-moderate SUI. The ICIQ-SF was used at baseline and to assess the improvement of SUI symptoms and any improvement in quality of life after treatment. Follow-up was done 3–6 months after treatment, which consisted of two treatment sessions with a vaginal erbium:YAG laser 21–28 days apart. Median ICIQ-SF scores were significantly lower post-treatment (11 and 3, respectively; *p* < 0.0001); 78.6% reported improvement of SUI and 38.1% reported a complete healing of SUI at follow-up; 66.7% of women reported high satisfaction with the treatment with 81.8% of sexually active women reporting an improvement in sexual gratification [10].

Pitsouni et al. [11] performed an observational study looking at the use of CO_2_ lasers in vaginal pathophysiology and the symptoms of GSM. The efficacy of the CO_2_ laser on lower urinary tract symptoms was assessed using the ICIQ-FLUTS, ICIQ-UI SF, urogenital distress inventory (UDI) and King's Health Questionnaire (KHQ). Participants received laser therapy once a month for 3 months and questionnaires were completed at baseline and 4 weeks after the last laser treatment (at 12-week follow-up). Fifty-three women were enrolled in the study. The was a significant reduction of the scores of the ICIQ-FLUTS, UDI-6, ICIQ-UI SF and KHQ at the 12-week follow-up compared with baseline. The authors concluded that the CO_2_ laser has a positive effect on the lower urinary tract resulting in treatment success of LUTS.

Another small pilot study of 22 women assessed the safety and efficacy of the intraurethral erbium:YAG laser for the treatment of type III stress incontinence (defined as intrinsic sphincter deficiency) [12]. Women were recruited based on having a Valsalva leak point pressure (VLPP) of 20–60 cmH_2_O. Pre-operative preparation included vitamin C and vitamin A for 3 months (as shown to increase collagen synthesis and a restorative process of the tissue at the level of the epithelium and lamina propria) and prophylactic antibiotics 2 h before intervention. Local estrogens were used when needed 2 weeks before the procedure. Patients received two laser sessions with a 3-week interval between the sessions. Follow-up was at 3 and 6 months after the first session. The frequency and severity of incontinence and impact on quality of life was assessed using the ICIQ-UI SF performed at baseline, 3- and 6-month follow-up. Patients with a score of 0 after treatment were considered cured; those with a one- or two-stage reduction in the ICIQ-UI SF compared with baseline were considered improved. Objective assessment of the severity of urinary leakage was performed with the standardised 1-h pad weight test. A clinically meaningful level of improvement was defined as a > 50% reduction of pad weight from baseline. Adverse events were also followed up at the 3- and 6-month time points. All 22 patients were classified as having severe SUI before treatment based on the ICIQ-UI SF. At 3 months after treatment 14 patents were cured and 4 improved. At the 6-month follow-up ten were cured and five were improved. There was no change in four patients at 3 months and seven patients at 6 months. Using the pad test, clinical improvement (as defined previously) was achieved in 18 (82%) patients at 3 months and 11 (50%) patients at 6 months. One patient reported pelvic pain and two patients reported dysuria; these adverse effects were transient, and all patients tolerated the procedure well. Despite the small numbers it was concluded that the intraurethral erbium:YAG laser seems to be a safe and efficacious treatment for patients with type III stress incontinence. No other studies using the intraurethral laser have been published.

Gambacciani et al. [13] recruited 205 post-menopausal women of which 114 had SUI. The ICIQ-SF was used to evaluate before and after treatment, which consisted of three vaginal erbium:YAG laser applications at 30-day intervals. Follow-up was for up to 24 months to assess for the long-term efficacy and acceptability of the vaginal erbium laser. The results demonstrated a significant decrease in ICIQ-SF scores from baseline to 12 months after the last application. However, at 18 and 24 months, ICIQ-SF scores were not significantly different from baseline (12.1 ± 2.5 at baseline, 9.3 ± 2.7 at 18 months and 9.9 ± 2.8 at 24 months). Interestingly, 96 of the 114 patients asked for a repeat laser treatment.

## Use of lasers in overactive bladder

Two studies have been published assessing the use of lasers in women with overactive bladder symptoms (OAB). Perino et al. [14] looked at 30 women with OAB [defined as ≥ 3-month frequency of micturition on average ≥ 8 times per 24 h and at least three episodes of urgency (grade 3 or 4), with or without incontinence, during a 3-day micturition diary period at baseline]. Treatment involved three sessions with a vaginal CO_2_ fractioned laser spaced over a 30-day period. Overactive bladder symptoms were assessed using the validated Overactive Bladder Questionnaire Short Form (OAB-Q SF). The study found a significant reduction in urinary frequency episodes and the number of urge episodes. There was also a significant decrease in the number of urge incontinence episodes in those women who suffered from it and a significant improvement in QoL from the OAB-q scores. Another small study, again with 30 patients, assessed the vaginal erbium:YAG laser on OAB symptoms, urinary incontinence and sexual function in women with USI [15]. Treatment consisted of two vaginal laser treatments, 4 weeks apart. OAB symptoms were assessed using the OABSS (overactive bladder symptoms score). Results showed that at 3-month follow-up OABSS symptom scores were significantly improved but this effect was not sustained at 12 months.

The following points should be noted from the available evidence in the literature about the use of lasers in urinary incontinence. The majority of studies have used vaginal erbium:YAG lasers with only two studies describing CO_2_ laser use. Only one study has assessed intraurethral use. All the studies are small with participants ranging from 22 to the largest study having 175 participants. Furthermore, no study has had a control or placebo group and all studies have been conducted in single centres. While the individual conclusions about the use of lasers in urinary incontinence are encouraging, the above deficiencies in the evidence must be considered.

## Use of lasers in genito-urinary syndrome of menopause (GSM)

GSM has been defined as including a range of symptoms related to a decline of circulating ovarian hormones, such as vaginal dryness, dyspareunia, recurrent urinary tract infections and urinary incontinence [4]. Laser therapy has been proposed as a treatment for GSM as it stimulates tissue repair and restores normal vaginal function [16].

First-generation laser treatment for GSM was done using the carbon dioxide (CO_2_) laser. This laser emits light at 10,600 nm [17]. Water absorbs the frequency of this light very well. Since water is a major constituent of mucosal tissues they are essentially vaporised to promote collagen formation.

Gaspar et al. [5] assessed the use of the vaginal CO_2_ laser in combination with local platelet-rich plasma (PRP) in 92 women with mild-to-moderate vaginal hypertrophy or atrophy. Patients were divided into two groups: the study group consisted of 40 patients (12 pre- and 20 postmenopausal) who underwent CO_2_ laser treatment (3 treatments, 44 days apart) with vaginal PRP injection and pelvic floor exercise in each session. The control group had 52 women (14 pre- and 30 postmenopausal) who received only PRP and pelvic floor exercise. Both groups were evaluated with a sexual health questionnaire and vaginal biopsies pre- and post-treatment. Those in the study group described a significant decrease in discomfort during sex compared with the control group. Histological analysis of the biopsies showed a significant increase in the fibrillar component of the extracellular matrix and fibroblast activity. In addition, there was a significant increase in the thickness of the vaginal epithelium and its glycogenic load after laser treatment. Two patients dropped out of the study group after two treatment cycles because of lack of effect and eight dropped out of the control group for the same reason (3 after 1 treatment cycle and 5 after 2 treatment cycles). Thirty per cent of patients described mild discomfort (pain or burning) at the time of laser treatment and up to 72 h after. The authors concluded that although beneficial effects of laser, PRP and pelvic floor exercise were seen immediately post-treatment in women with urovaginal atrophy, more data are needed to better address the use of these procedures.

This initial study was followed by a 12-week pilot study assessing the use of vaginal fractional CO_2_ lasers for vulvovaginal atrophy [18]. Symptoms were analysed before and after three sessions (1 session per month) of the fractionated CO_2_ laser using the Vaginal Health Index Score (VHIS), intensity of vulvo-vaginal atrophy (VVA) symptoms using a 10-point VAS and the Short Form-12 (SF-12) to assess the physical and mental component. Forty-nine women completed the study. The VHIS improved significantly after each laser application compared with baseline. Each VVA symptom (vaginal dryness, vaginal burning, vaginal itching, dyspareunia and dysuria) significantly improved at the 12-week follow-up compared with baseline. In addition, no adverse events related to the fractional CO_2_ laser were recorded throughout the study period. This study was limited by the small sample size, short duration and lack of long-term follow-up, and absence of a comparator.

The same study group further investigated the effects of vaginal CO_2_ lasers on sexual function and overall satisfaction with sexual life in 77 post-menopausal women with VVA treated with three laser sessions at 30-day intervals. The Female Sexual Function Index (FSFI) and SF-12 were used at baseline and at 12-week follow-up. There was a significant increase in total scores and each individual domain of the FSFI at the 12-week follow-up compared with baseline. In addition of the 20 women who were not sexually active because of VVA, 85% regained normal sexual activity at the 12-week follow-up [19].

A replication of the above study was conducted by Perino et al. [20] in post-menopausal women with VVA symptoms. Forty-eight patients were enrolled and treated with fractional CO_2_ laser via a vaginal probe. All patients underwent a complete cycle of three treatment sessions spaced over 30 days. At baseline and 30 days post last treatment, the vaginal status of the women was evaluated using the Vaginal Health Index Score (VHIS) and subjective intensity of VVA was evaluated using a VAS. This study again demonstrated significant improvement in the VHIS after laser treatment and 91.7% of patients were satisfied or very satisfied with the treatment. No adverse events due the CO_2_ laser were reported.

Pitsouni et al. performed an observational study looking at the use of CO_2_ lasers in vaginal pathophysiology and the symptoms of GSM. The effect on lower urinary tract symptoms has been described above; however, the primary outcomes of this observational study were the vaginal maturation value and VHIS [11]. Secondary outcomes included symptoms of GSM and the female sexual function index (FSFI). Participants received vaginal CO_2_ laser therapy once a month for 3 months and questionnaires were completed at baseline and 4 weeks after the last laser treatment (at 12-week follow-up). Fifty-three women (postmenopausal with 1 or more symptoms of GSM with moderate to severe intensity and clinical signs of GSM) were enrolled in the study. The VHIS significantly improved at 12-week follow-up compared with baseline. In addition, the severity of GSM significantly decreased while sexual function increased significantly.

Sokol et al. [21] carried out the first US study looking at the use of the CO_2_ laser for the treatment of VVA, evaluating the safety and efficacy. Women presenting with GSM were enrolled and assessments performed at baseline and 3 months after the final treatment. Women received three vaginal laser treatments, 6 weeks apart. Visual analogue scales were used to assess pain, burning, vaginal itching, vaginal dryness, dyspareunia and dysuria; VHIS, FSFI and SF-12 questionnaires were also completed. Of the 30 women recruited, 27 completed the study. For all six symptoms of VVA there was a statistically significant improvement in symptoms at follow-up compared with baseline. There was also a statistically significant improvement in mean VHIS scores, FSFI scores and at follow-up compared with baseline scores. There was a non-significant improvement in SF 12 scores assessing physical and mental health at baseline and at 12-week follow-up. Adverse events included mild to moderate pain lasting 2–3 days in two women and two women reported minor bleeding lasting < 1 day; however, none of these women discontinued treatment. Twenty-three women were followed up at 1-year post-treatment [22]. All VVA symptoms continued to be significantly better compared with baseline except dysuria. VHIS and FSFI scores also showed statistically significant improvement at 1 year compared with baseline. Ninety-two per cent of women were satisfied or extremely satisfied at 1-year post-treatment. Although encouraging results, the small numbers and lack of placebo group mean these findings need cautious interpretation.

The long-term safety, feasibility and efficacy of CO_2_ lasers in women with GSM were assessed by Behnia-Willison et al. [23]; 102 women with symptomatic GSM were treated with a vaginal fractional CO_2_ laser. These women were either not responding or unable to take conventional oestrogen therapy for postmenopausal symptoms. All eligible patients received three treatments at ≥ 6-week intervals with follow-up for 12 months after the first treatment. Patients were asked to complete the Australian pelvic floor questionnaire. Intensity of GSM symptoms (vaginal dryness, vaginal dyspareunia, vaginal tightness, prolapse symptoms, bladder function and urge and stress incontinence) was recorded using measures of frequency and severity. Data were collected at baseline before the first treatment, between 2 and 4 months from initial treatment and between 12 and 24 months after the initial laser treatment. There was a statistically significant improvement in GSM symptoms following treatment with the median severity score decreasing from 2 pre-treatment to 0 post-treatment. There was an increase in patients reporting ‘normal’ sexual function from baseline (24.1%) to 12–24-month follow-up (63.6%). In addition, the percentage of patients reporting ‘never’ for painful intercourse increased from 13.2% at baseline to 34.2% at 12-24-month follow-up. One limitation of this study was that less than half the patients were followed up after the third treatment to the 12–24-month follow-up. Furthermore, there was no control group.

Gambacciani et al. [6] evaluated the short-term effectiveness and acceptability of the vaginal erbium:YAG laser for the treatment of GSM. This was a pilot, prospective, longitudinal study performed in a single centre in postmenopausal women suffering from GSM. Inclusion criteria were presence of GSM in healthy postmenopausal women (at least 1 year since the last menstrual period or bilateral oophorectomy) with plasma gonadotropin and estradiol levels in the post-menopausal range and a negative smear test. Women were excluded if they had vaginal lesions, scars, active or recent genito-urinary infection, abnormal uterine bleeding, had used lubricants within 30 days of the study, history of photosensitive disorder or use of photosensitive drugs, grade 2–3 genital prolapse, serious or chronic condition affecting compliance, or use of hormones or other medicines to relieve menopausal symptoms in the 12 months before the study. Forty-five patients were treated with vaginal erbium:YAG lasers in SMOOTH mode in the outpatient setting without any specific preparation, anaesthesia or post-operative medications. Before the procedure the vagina was cleaned with disinfectant solution and dried with a swab. Patients had three laser treatments (1 every 30 days). They had a screening visit 2–4 weeks before the first procedure and were followed up at 4, 12 and 24 weeks from the last laser application. The active control group consisted of 25 postmenopausal women treated with 1 g vaginal gel containing 50 μg estriol twice weekly for 3 months. GSM was evaluated with subjective (visual analogue scale, VAS) and objective (Vaginal Health Index Score, VHIS) measures at each visit. Results were analysed in 43 patients in the laser group and 19 women in the estriol group. Laser treatment induced a significant decrease of VAS of both vaginal dryness and dyspareunia (*p* < 0.01). This decrease in VAS was also seen in the estriol group with no significant difference noted during the treatment period between the two groups. However, the VAS scores in the estriol group measured after 24 weeks were significantly higher compared with the laser group. VHIS scores increased significantly in both groups (laser and estriol) from basal values during treatment (10.6 ± 3.6 and 11.2 ± 2.8, respectively, to 20.1 ± 1.8 and 22.4 ± 4.0, respectively, *p* < 0.01) but decreased at the 12- and 24-week follow-up. The values in the estriol group were significantly different from corresponding values in the laser group. The vaginal erbium:YAG laser was well tolerated with < 3% of patients discontinuing treatment because of adverse effects (unacceptable to patient, burning sensation reported 3 days after first application). The authors concluded that this pilot study showed the erbium:YAG laser induces a significant improvement in GSM, including vaginal dryness and dyspareunia. The effect appeared to be maintained throughout the study period up to the 24-week follow-up whereas a reduction in efficacy was noted in the estriol group.

Gaspar et al. [24] assessed the efficacy and safety of the vaginal erbium:YAG laser for GSM and compared it with topical estriol treatment. The estriol group (*n* = 25) received 0.5 mg estriol ovules for 8 weeks (daily for 2 weeks, then 3 times a week for 2 weeks, then twice a week for 4 weeks) and the laser groups (*n* = 25) received pre-treatment of 0.5 mg estriol ovules three times a week for 2 weeks (to hydrate the vaginal mucosa) and then received three laser sessions (1 every 3 weeks) over the 8-week course. Outcome data included patient discomfort and treatment tolerability as well as potential adverse effects. VAS analysis of symptoms was recorded up to 18 months post-treatment. Biopsies for histological examination were taken from six patients in each group before treatment and at 1, 3, 6 and 12 months post-treatment. There was a significant reduction in all GSM symptoms in both groups at 6 months and the effect of the laser treatment remained statistically significant at 12- and 18-month follow-up. Four per cent of patients in the laser group reported mild to moderate pain and transient oedema.

The same group [25] also specifically looked at treatment of urinary symptoms of GSM using the intraurethral erbium:YAG laser. Twenty-nine postmenopausal women were included in the study. Inclusion criteria were patients diagnosed with GSM, having < 5% vaginal superficial cells in the cytology, vaginal pH > 5 with urinary symptoms of GSM (frequency, dysuria and urgency) and impaired continence due to urethral atrophy. All patients received two intraurethral erbium:YAG laser sessions with a 3-week interval between sessions. Therapeutic efficacy was assessed using the ICIQ-SF, 1-h pad test and VAS scores. Follow-up was at 3 and 6 months with the occurrence of any adverse events. VAS values for urinary symptoms significantly decreased from baseline at the 3- and 6-month follow-up. Average ICIQ-UI and 1-h pad test values at baseline were 13 and 42 (gh^−1^), respectively. Values at the 3-month follow-up were 5.2 and 16, respectively, and at the 6-month follow-up were 8.1 and 23 (statistically different from baseline values, *p* < 0.0005). Observed side effects included dysuria and minimal haematuria (transient) and one patient had a urinary tract infection requiring antibiotics. The authors concluded that their findings suggest the intraurethral erbium:YAG laser is an efficacious and safe modality for treatment of urinary symptoms of GSM but that larger randomised trials are needed the better assess the long-term effect of this procedure.

Long-term data on vaginal erbium:YAG laser use for treatment of GSM followed up 205 postmenopausal women before and after treatment (consisting of 3 laser applications every 30 days) throughout 24 months using the VAS and VHIS [13]. The active control group consisted of post-menopausal women treated with either estriol gel 50 mg twice weekly or non-hormonal established therapies (such as lubricants or moisturisers) for 3 months. This study found that although VAS scores for vaginal dryness and dyspareunia were significantly different from baseline after the second and third laser treatments and during the first 12 months of observation, the values measured after 18 and 24 months from the last laser application were not significantly different from basal values (VAS for basal vaginal dryness and at 24 months: 8.3 ± 1.5 and 7.0 ± 1.0, respectively, VAS for basal dyspareunia and at 24 months: 8.5 ± 1.3 and 8.2 ± 2.4, respectively). In addition, only 66 of the 205 laser patients completed 24 months of follow-up with 174 patients having repeat laser treatment from 6 months onwards. No adverse events related to the procedure were recorded throughout the study period. The authors concluded that laser treatment improved GSM symptoms for 12 months after the last laser application while the effects seem to vanish afterwards, highlighting the need to investigate how soon after repeat laser treatment can be offered for GSM.

In 2015 Gambiacciani et al. published their protocol for the VELAS study. The protocol described an international multicentre observational study on GSM and SUI evaluating the effect of three laser applications in 1500 post-menopausal women with subjective and objective symptom evaluation at baseline and for up to 1 year post-treatment [26]. No further data on this study can be found in the literature to date.

## USE of lasers in female prolapse

The non-ablative erbium:YAG laser when used in SMOOTH mode delivers precisely controlled, sequentially packaged bursts of long pulses. Its thermal effects on soft tissue result in deep collagen remodelling and new collagen synthesis [16]. It has been suggested that vaginal erbium lasers can be used in disorders of diminished pelvic floor support such as pelvic organ prolapse (POP) or vaginal relaxation syndrome (VRS, where the relaxing of the vaginal wall leads to physical and psychological problems mainly related to decreasing sexual satisfaction). Gaviria et al. [27] conducted a pilot study on 21 women with VRS and evaluated the safety and efficacy of vaginal erbium:YAG treatment. All patients received two treatment sessions with a 15–30 interval between sessions. Assessment was with a specifically designed laser vaginal tightening questionnaire, POP-Q measurement and the PISQ-12 questionnaire. Twenty of the 21 patients reported a significant improvement of their vaginal tightness and better sex after treatment. Five patients had stage 1–3 prolapse and all five reported improvement after laser treatment. There were no adverse effects and minimal patient discomfort.

Another study recruited 30 postpartum females with VRS [3]. These women were divided into two groups, A and B. Both groups were treated for four sessions at 1- to 2-weekly intervals with vaginal erbium:YAG via 90° and 360° scanning scopes. In group A the first two sessions were performed with the 360° scope and the final two with the 90° scope in multiple micro-pulse mode, 1.7 J delivered per shot, three multishots and three passes per session. Group B underwent multiple micro-pulse mode treatments with the 90° scope in all four sessions (same parameters as in group A) and then during the final two sessions an additional two passes/session were delivered with the 360° scope, long-pulse mode, 3.7 J delivered per shot. Perineometer assessments were performed at baseline and at 2 months post-treatment for vaginal tightness. Histological specimens were taken at baseline and at 2 months post-procedure. Subjective satisfaction with vaginal tightening was assessed together with improvement in sexual satisfaction. All subjects successfully completed the study with no adverse events. Significant improvement in vaginal wall relaxation was seen in all subjects at 2 months post-procedure based on the perineometer values and on the partners’ input for vaginal tightening (76.6%) and for sexual satisfaction as assessed by the subjects themselves (70.0%). The histological findings suggested better elasticity of the vaginal wall with tightening and firming. The authors concluded that both regimens of vaginal erbium:YAG laser treatment for VRS produced significant improvement in vaginal relaxation with no adverse effects.

A pilot study presented at IUGA in 2013 evaluated vaginal erbium:YAG laser use as a technique for prolapse reduction [28]. Twenty-eight women with grade II–IV cystoceles (Baden-Walker classification) received between one and three treatment sessions with 2-month intervals. Follow-up was at 2.4 and 6 months. At baseline patients underwent examination and post-void residual measurement. These were also done at follow-up in addition to enquiring about any procedure-related adverse effects; 96.4% of patients had their prolapse reduced by one grade, 42.9% by two grades and 7.1% by three grades. There was a significant reduction in post-void residuals. There were no treatment-related adverse effects and the majority of patients were either satisfied or very satisfied.

Another study assessed 83 patients with grade II–IV cystoceles and follow-ups were performed at 2, 6, 12, 24 and 36 months after the first laser session [29]. Patients were treated with a vaginal erbium:YAG laser (between 2 and 7 sessions with a 2-month interval between each session). Sixty-seven women were followed up for a longer period and included in this study. The average prolapse grade before the treatment was 2.36 ± 0.62 and was significantly (*p* < 0.001) reduced already after the first session (to 1.5 ± 0.79). The prolapse continued to improve with sessions, reducing to 0.94 ± 0.78 after 6 months and 0.86 ± 0.78 after 12 months. Average grade at 24 months was 1.13 ± 1.02 and at 36 months 0.89 ± 0.79. Treatment discomfort was very low (average score of 0.4 on a 10-point VAS) and patient satisfaction high (median level of 4 on a scale of 1–5). No adverse effects of this treatment were reported. The authors concluded that vaginal erbium:YAG laser treatment for higher-grade cystoceles demonstrated good efficacy in the improvement of cystoceles and minimal patient discomfort during the treatment, with no adverse effects. The improvement lasted at least 12 months and for many patients even longer up to 36 months.

## Discussion

The evidence published on the use of vaginal lasers is for their use in urinary incontinence, GSM and pelvic organ prolapse. Although not covered in this article, there is moderate evidence to suggest that laser treatment results in histological change within the vaginal tissues and collagen ‘remodelling’ [30]. The data on its use in stress urinary incontinence comprise short-term observational studies. Participants varied from 19 to 205 women. Treatment response was assessed with validated questionnaires and showed favourable outcomes in terms of improvement of symptoms but only one study followed patients up for 24 months [13]. None of the studies had a control or placebo group.

In investigating its use in GSM one study compared laser/PRP and pelvic floor exercises and PRP and pelvic floor exercises alone and assessed sexual function. Although the use of lasers seemed to demonstrate a significant improvement in sexual function compared with the non-laser group, the sample size was small and follow-up was short term [5]. Another study compared vaginal laser treatment with topical estriol and topical estriol alone [24]. Follow-up was longer in this study and at 18 months post-treatment the laser group had a statistically significant reduction in GSM symptoms. Again, this suggests that lasers may be beneficial, but robust evidence is lacking because of the small sample size and single-centre study design. Observational studies looking at laser treatment and GSM with follow-up ranging from 12 weeks post-treatment to 12 months show significant improvement in GSM symptoms, but the lack of numbers and comparative groups diminishes the quality of the evidence. One study compared laser with estriol gel treatment and followed patients up for 24 months, but the beneficial effects of the laser did not seem to be sustained beyond 12 months after the last laser application [13].

There are minimal published data on the use of lasers in treating female pelvic organ prolapse (POP) and vaginal relaxation syndrome (VRS). Two small studies on the use of lasers in VRS comprising 51 women in total showed patient-reported improvements in sexual experience after laser treatment but follow-up was short term. Its use in POP has been described in women with grade II to IV cystoceles and follow-up at 12 months has demonstrated an improvement in prolapse grade, with some patients sustaining the effect at 36 months [29].

The majority of studies did not state that previous vaginal surgery was one of their exclusion criteria; however, the role of lasers in scarred or fibrosed tissue as opposed to virgin tissue may impact its efficacy.

While the use of lasers to treat these conditions may seem appealing, the lack of good-quality evidence in the form of multi-centre randomised placebo-controlled trials is concerning. A recent publication from the US Food and Drug Administration has highlighted that although certain companies are marketing laser devices for ‘vaginal rejuvenation’ the term itself lacks clarity. The safety and effectiveness of these laser devices have not been established. Use of lasers may lead to serious adverse events such as vaginal burns, scarring, dyspareunia and chronic pain. There are few reports of adverse events in the literature, and the sample sizes are small; hence, minimal reassurance can be taken from them.
